# Full-length isoform concatenation sequencing to resolve cancer transcriptome complexity

**DOI:** 10.1186/s12864-024-10021-x

**Published:** 2024-01-29

**Authors:** Saranga Wijeratne, Maria E. Hernandez Gonzalez, Kelli Roach, Katherine E. Miller, Kathleen M. Schieffer, James R. Fitch, Jeffrey Leonard, Peter White, Benjamin J. Kelly, Catherine E. Cottrell, Elaine R. Mardis, Richard K. Wilson, Anthony R. Miller

**Affiliations:** 1 The Steve and Cindy Rasmussen Institute for Genomic Medicine, Abigail Wexner Research Institute at Nationwide Children’s Hospital, 575 Children’s Crossroad, Columbus, OH 43215 USA; 2 Department of Pediatrics, The Ohio State University College of Medicine, Columbus, OH USA; 3 Department of Pathology, The Ohio State University College of Medicine, Columbus, OH USA; 4 Department of Neurosurgery, Nationwide Children’s Hospital, Columbus, OH USA; 5 Department of Neurosurgery, The Ohio State University College of Medicine, Columbus, OH USA

**Keywords:** Long-read RNA sequencing, Concatenation, Isoform discovery, Tumor transcriptome

## Abstract

**Background:**

Cancers exhibit complex transcriptomes with aberrant splicing that induces isoform-level differential expression compared to non-diseased tissues. Transcriptomic profiling using short-read sequencing has utility in providing a cost-effective approach for evaluating isoform expression, although short-read assembly displays limitations in the accurate inference of full-length transcripts. Long-read RNA sequencing (Iso-Seq), using the Pacific Biosciences (PacBio) platform, can overcome such limitations by providing full-length isoform sequence resolution which requires no read assembly and represents native expressed transcripts. A constraint of the Iso-Seq protocol is due to fewer reads output per instrument run, which, as an example, can consequently affect the detection of lowly expressed transcripts. To address these deficiencies, we developed a concatenation workflow, PacBio Full-Length Isoform Concatemer Sequencing (PB_FLIC-Seq), designed to increase the number of unique, sequenced PacBio long-reads thereby improving overall detection of unique isoforms. In addition, we anticipate that the increase in read depth will help improve the detection of moderate to low-level expressed isoforms.

**Results:**

In sequencing a commercial reference (Spike-In RNA Variants; SIRV) with known isoform complexity we demonstrated a 3.4-fold increase in read output per run and improved SIRV recall when using the PB_FLIC-Seq method compared to the same samples processed with the Iso-Seq protocol. We applied this protocol to a translational cancer case, also demonstrating the utility of the PB_FLIC-Seq method for identifying differential full-length isoform expression in a pediatric diffuse midline glioma compared to its adjacent non-malignant tissue. Our data analysis revealed increased expression of extracellular matrix (ECM) genes within the tumor sample, including an isoform of the Secreted Protein Acidic and Cysteine Rich (*SPARC*) gene that was expressed 11,676-fold higher than in the adjacent non-malignant tissue. Finally, by using the PB_FLIC-Seq method, we detected several cancer-specific novel isoforms.

**Conclusion:**

This work describes a concatenation-based methodology for increasing the number of sequenced full-length isoform reads on the PacBio platform, yielding improved discovery of expressed isoforms. We applied this workflow to profile the transcriptome of a pediatric diffuse midline glioma and adjacent non-malignant tissue. Our findings of cancer-specific novel isoform expression further highlight the importance of long-read sequencing for characterization of complex tumor transcriptomes.

**Supplementary Information:**

The online version contains supplementary material available at 10.1186/s12864-024-10021-x.

## Background

A gene can encode multiple mature mRNA transcripts through the process of alternative splicing, whereby differential exon retention modulates the final structure of the transcript isoform and resulting protein [1]. These proteins may have distinct functions, expanding diversity within the cellular proteome [2]. While alternative splicing is integral to basic functions like cellular homeostasis, dysregulation of RNA splicing can promote tumorigenesis as specific isoforms are linked to cancer progression, metastasis, and therapeutic resistance [3–5]. These findings underscore the importance of accurate detection and identification of isoforms within the cancer transcriptome to aid in a better understanding of cancer biology and may have diagnostic and/or prognostic implications for patient care.

Next-generation sequencing (NGS) methodologies have proven valuable by enabling an unbiased study of the transcriptome, which in the setting of cancer, can specifically allow for detection of actionable findings, such as aberrant gene expression, gene fusions, and other structural variation [6–10]. Short-read NGS technologies provide sufficient read depth for assessment of gene expression at a relatively low-cost point compared to other methodologies [11] but are confined to limited sequence lengths (< 300 bp reads) to maintain base-calling accuracy. [12, 13] Most human protein coding transcripts are longer than the short-read length, requiring RNA-sequencing (RNA-Seq) read assembly to identify expressed transcript isoforms [14, 15]. Individual short reads that fail to span successive splice sites or originate from regions of high homology or genomic complexity can negatively impact accurate transcript assembly and hinder this identification [16].

Long-read sequencing platforms, however, permit full-length RNA isoform sequencing (e.g., Pacific Biosciences (PacBio) Iso-Seq protocol). The Iso-Seq protocol requires no computational assembly of reads, as the method preserves both the full-length expressed exonic order and orientation of each transcript. As such, Iso-Seq data represents native transcripts that can accurately represent novel isoforms [17–22]. The improvement in characterization of expressed isoforms by full-length long-read sequencing comes at the expense of comparatively lower read output per instrument run, which can limit the detection of moderate to lowly-expressed isoforms [23]. To ameliorate this limitation, and to improve overall unique isoform detection, we pursued development of transcriptome-based expression methods using long-read sequencing aimed at increasing the total number of sequenced transcript isoforms in a single instrument run.

The PacBio Sequel IIe instrument and a single 8 M SMRT Cell, on average, yields up to four million high fidelity (HiFi) reads using circular consensus sequencing (CCS) with read accuracy equivalent to short-read sequencing technology [24]. The circular structures comprising the PacBio library (SMRTbell) coupled with the instrument run time, enables multiple polymerase passes around each template, with each pass generating a subread. Subsequent analysis collapses these subreads to form a highly accurate (“HiFi”) read. An optimal SMRTbell library insert of 15,000 bp requires only 10 polymerase passes to achieve 99.9% read accuracy [24]. However, the Iso-Seq method produces smaller SMRTbell inserts, resulting in an excessive number of circular passes, and effectively wasting sequencing output. Protocols have been designed to optimize sequencing output by concatenating individual transcripts prior to the SMRTbell adapter ligation step in library construction [25, 26]. These reported methods, however, were designed to concatenate molecules smaller than 870 bp, minimizing the efficacy of capturing full-length isoforms. Introduction of the Multiplexed Arrays Sequencing (MAS-seq) workflow, [27] a technique for programmable concatenation of cDNA molecules, provided a unique approach to boost output on the PacBio platform. Here, individual barcoded cDNA transcripts are ligated into concatenated molecules (up to 15-mer) prior to SMRTbell generation. The utility of the MAS-seq protocol was initially demonstrated using 10xGenomics single cell cDNA, yielding approximately 40 million reads from a single 8 M SMRT Cell [27].

In this work, we present a method for concatenation of cDNA molecules generated from the combination of the PacBio Iso-Seq and MAS-seq protocols. Our method, PacBio Full-Length Isoform Concatemer Sequencing (PB_FLIC-Seq), is designed to more fully characterize the transcriptome of a sample, including moderate to lowly expressed isoforms and to take advantage of the long read lengths of the platform to identify full-length transcript isoforms. The PB_FLIC-Seq workflow is unique compared to the aforementioned methods, in that cDNA molecules are size-selected prior to concatemer formation to reduce any potential concatenation bias against longer transcripts (> 2,000 bp). To demonstrate the potential of this method we first used a commercial reference RNA standard with known synthetic isoform complexity and demonstrated a 3.4-fold increase in read output relative to the same sample processed using the Iso-Seq protocol. In addition to improved output, we demonstrated improved recall and reproducibility in the detection of the known synthetic isoforms using PB_FLIC-Seq. Subsequently, we applied the PB_FLIC-Seq method to RNA isolated from a pediatric diffuse midline glioma sample and its adjacent non-malignant tissue, illustrating the identification of differential isoform expression in the cancer sample.

## Results

### Evaluation of the PB_FLIC-Seq workflow using a commercial reference standard RNA (HBR_SIRV)

**Fig. 1:**
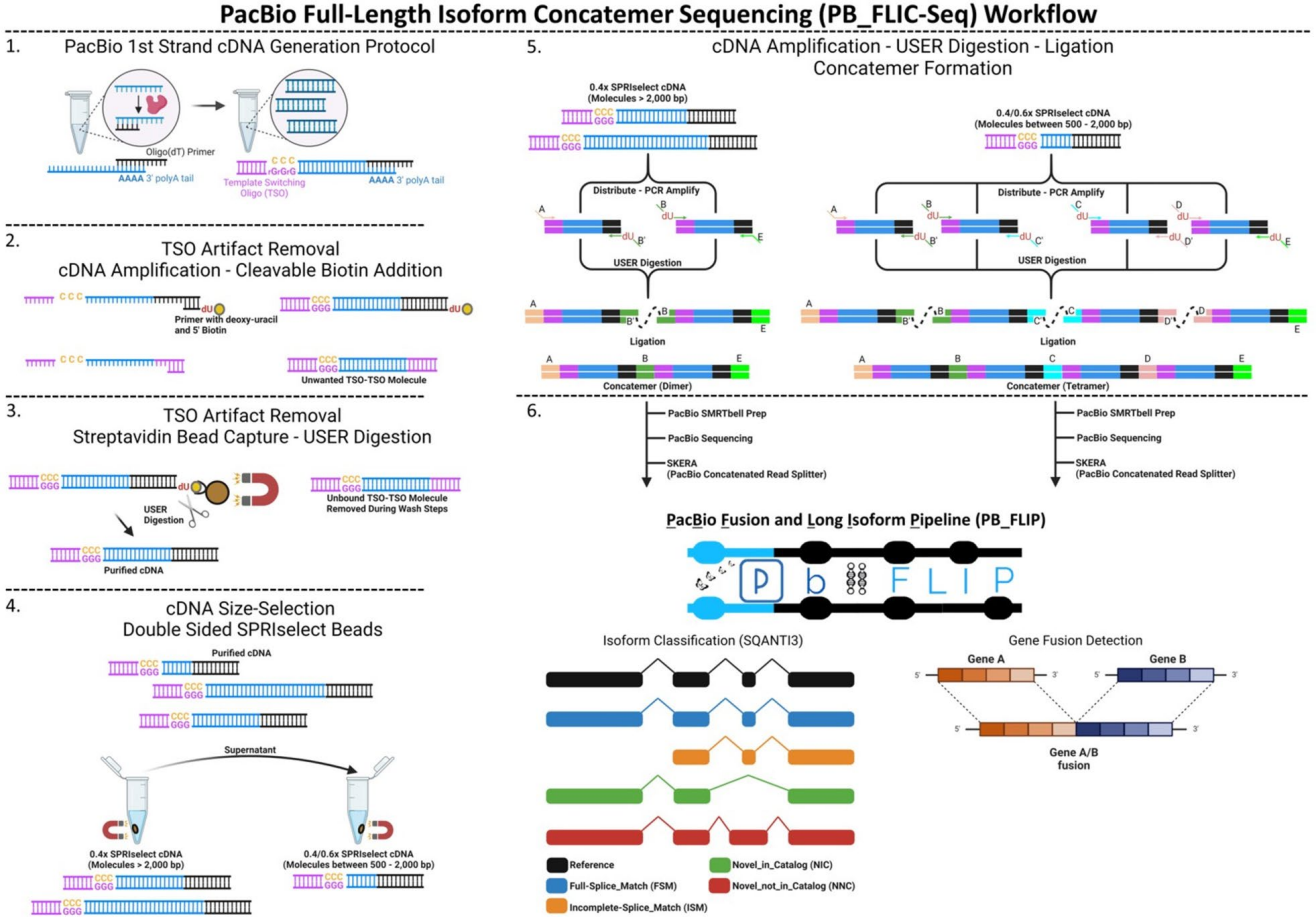
PacBio Full-Length Isoform Concatemer Sequencing (PB_FLIC-Seq) overview. 1) First-strand cDNA generation using oligo(dT) priming. 2) Biotin addition via cDNA amplification for pending template switching oligo (TSO) artifact removal. 3) Bead based capture to remove TSO artifacts. 4) cDNA size-selection prior to concatemer formation. 5) PCR using primers containing known reverse-complement barcodes with uracil incorporation ("dU”), followed by USER digestion and ligation to form the concatemer array. 6) Samples undergo PacBio SMRTbell prep, sequencing, and PacBio primary analysis including SKERA for read splitting. Deconcatenated reads are used as input into the PacBio Fusion and Long Isoform Pipeline “PB_FLIP” [17] for isoform characterization and gene fusion detection

We compared data from PacBio sequencing of three replicates of a PB_FLIC-Seq library (Fig. 1) prepared with the HBR_SIRV commercial reference RNA (“HBR_SIRV_Con”) to a previously published HBR_SIRV Iso-Seq dataset (“HBR_SIRV_Non") [17].

The PacBio SMRT Link analysis output calculated the total number of HiFi reads sequenced for each sample and, demonstrated differences between the HBR_SIRV_Non (2,501,016 reads) and the HBR_SIRV_Con samples (3,184,642) reads (Additional file 1). For the HBR_SIRV_Con samples, the PacBio SKERA software analyzed the known barcodes at each end of individual cDNA molecules to enumerate the concatenated HiFi read (“segmented reads” (s-reads)), demonstrating that the HBR_SIRV_Con samples generated on average 8,432,861 s-reads; a 3.4-fold increase over HBR_SIRV_Non (2,501,016 s-reads).

We implemented a cDNA size-selection step prior to concatemer formation to reduce intermolecular ligation bias (see methods), as ligation reactions are driven in part by the kinetics of end-joining with short cDNA molecules having enhanced ligation efficiency [28]. In separate reactions and through using barcode-directed ligation, cDNA molecules > 2,000 bp were concatenated into multiples of two molecules (“dimer”), while cDNA size-selected between 500 – 2,000 bp were concatenated into multiples of four molecules (“tetramer”) before final pooling of each ligation reaction to construct HBR_SIRV_Con SMRTbell libraries. Of the 8,432,861 s-reads, 2,655,797 were generated from the dimer and 5,777,064 from the tetramer molecules. The efficiency in generating the full concatemer formation was on average 77.7% and 72.7% for the dimer and tetramer molecules respectively, as calculated by SKERA (Additional file 1).

### SIRV isoform recall is improved with PB_FLIC-Seq

**Fig. 2:**
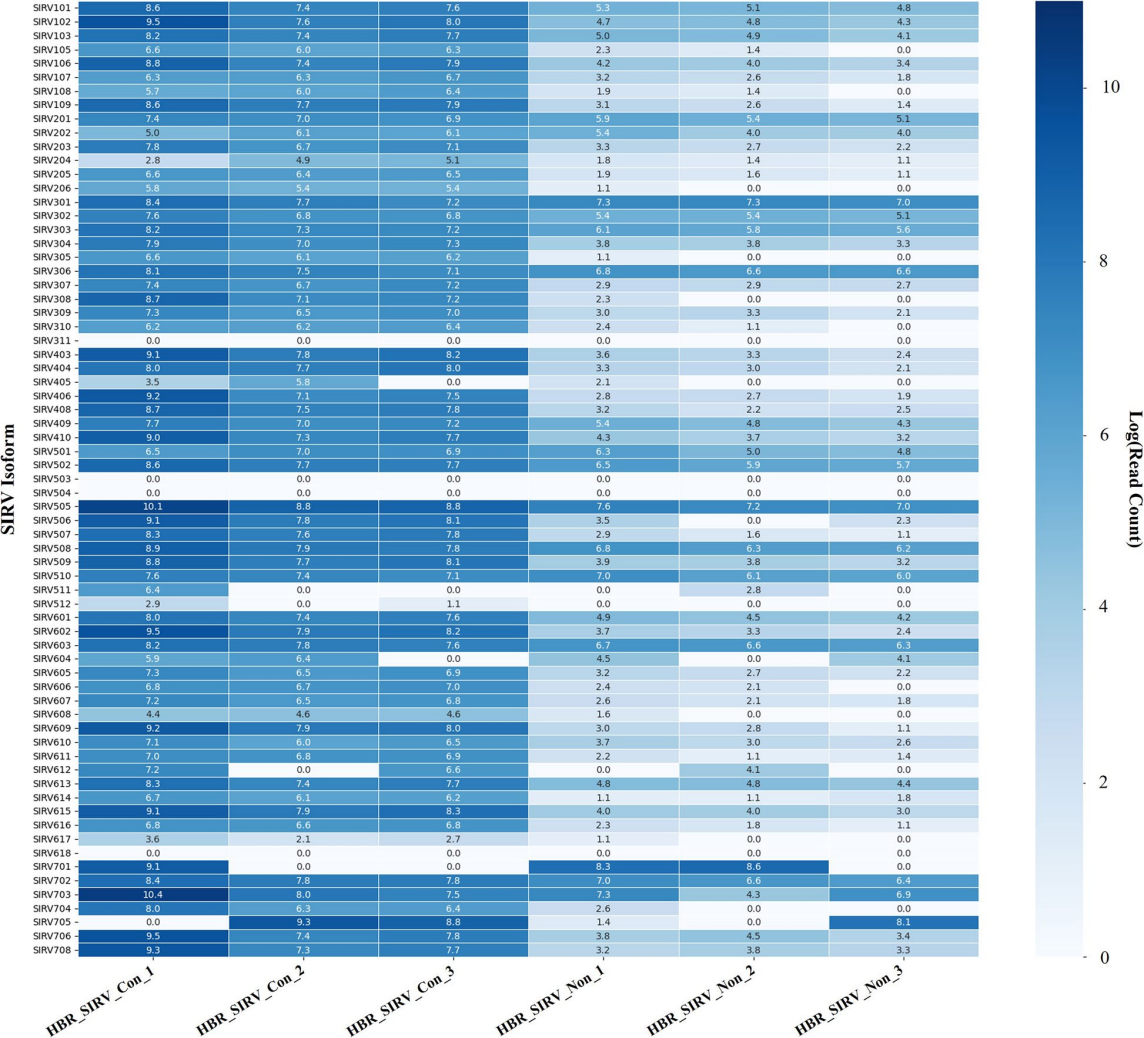
SIRV isoform recall between sample prep methods. Heatmap illustrating detected full-length Spike-In RNA Variants “SIRV” isoform read count for the 69 known SIRV isoforms (y-axis) spiked into the HBR_SIRV_Con and HBR_SIRV_Non samples (x-axis). The samples include Human Brain Reference RNA mixed with SIRV prepared with the PacBio Full-Length Isoform Concatemer Sequencing workflow “HBR_SIRV_Con” or prepared with a non-concatemer Iso-Seq workflow “HBR_SIRV_Non”. For determining percent SIRV recall, a SIRV isoform is considered detected if supported by at least one Circular Consensus Sequencing “CCS” read

Recall and reproducibility of the PB_FLIC-Seq workflow for isoform detection accuracy were assessed by measuring the representation of the 69 known synthetic SIRV isoforms in the HBR_SIRV_Con and HBR_SIRV_Non datasets. An average of 165,457 full-length SIRV isoform reads were identified in each of the HBR_SIRV_Con samples. Of the 69 SIRV isoforms, each HBR_SIRV_Con replicate averaged a percent recall of 89.9% with 64, 61, and 61 SIRV isoforms detected, which collectively represented 65 of 69 SIRV isoforms (Fig. 2; Additional file 2). Of the identified 65 SIRV isoforms, 58 (89.2%) of the isoforms were detected in all three sample replicates. The HBR_SIRV_Non libraries, by comparison, had fewer full-length SIRV isoform reads (average of 14,434), resulting in fewer SIRV isoforms detected (62, 54 and 50 SIRV isoforms); with an average recall of 80.2%. In total, 64 unique SIRV isoforms were detected between the HBR_SIRV_Non replicates, with 47 (73.4%) SIRV isoforms observed in all three samples.

### Evaluating transcriptome diversity of HBR_SIRV samples

**Fig. 3:**
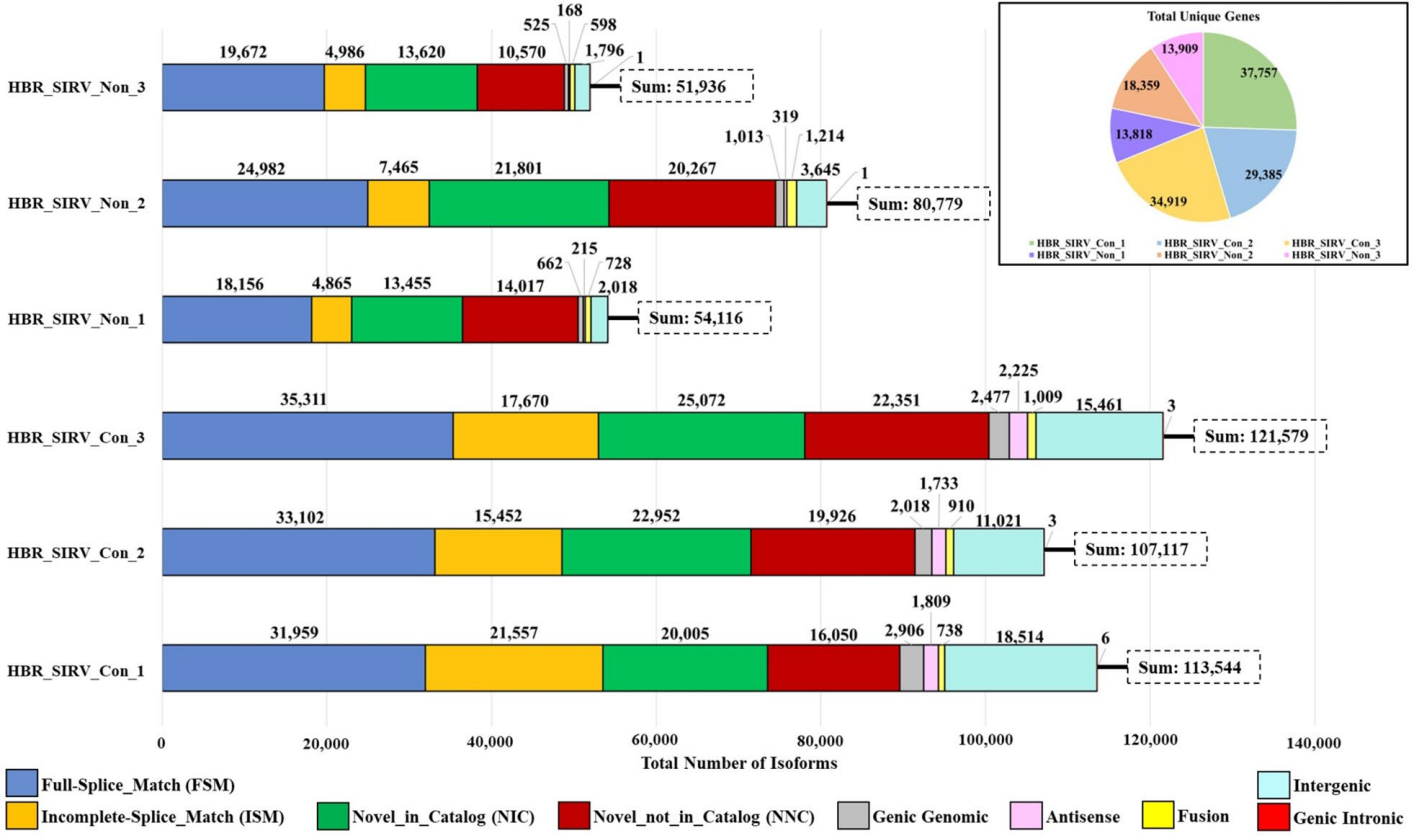
SQANTI3 classification for detected isoforms between sample prep methods. The samples include Human Brain Reference RNA mixed with Spike-In RNA Variants “SIRV” prepared with the PacBio Full-Length Isoform Concatemer Sequencing workflow “HBR_SIRV_Con” or prepared with a non-concatemer Iso-Seq workflow “HBR_SIRV_Non”. The pie graph illustrates the number of detected unique genes for the HBR_SIRV_Non and HBR_SIRV_Con samples, in which unique genes are defined as reference-based annotated genes and those genes curated from sample specific novel isoforms. The bar graph illustrates categorization of identified isoforms using the SQANTI3 classifier. SQANTI3 classes include Full-Splice Match (FSM; isoform aligns completely to reference), Incomplete-Splice Match (ISM; isoform partially aligns to a known reference transcript), Novel_in_Catalog (NIC; isoform containing novel combinations of known donor and acceptor splice sites), Novel_not_in_Catalog (NNC; isoforms containing at least one unannotated splice site), Genic Genomic (isoform containing partial exon and intron/intergenic overlap), Antisense (isoform containing overlap with complementary strand of an annotated transcript), Fusion (isoforms spanning two annotated loci), Intergenic (isoform aligns completely outside of annotated gene), and Genic Intronic (isoform aligns entirely within an annotated intron)

Transcriptome diversity was evaluated for the HBR_SIRV libraries using the SQANTI3 analysis classifier [29] to assess changes in detected isoform complexity from the PB_FLIC-Seq data. SQANTI3 outputs include the number of unique genes with detected expressed isoforms, including genes curated from sample-specific novel isoforms, as well as isoform classification based on identified splice junctions. As depicted in Fig. 3's pie chart, this analysis revealed a notable average increase of 2.2-fold in the total number of unique genes identified in the HBR_SIRV_Con datasets (with an average of 34,020 unique genes) compared to the HBR_SIRV_Non datasets (with an average of 15,362 unique genes). Furthermore, the bar plot in Fig. 3, illustrating the number of isoforms detected per sample, shows that the average total number of isoforms identified in the HBR_SIRV_Con datasets (amounting to 114,808 isoforms) increased by 1.8-fold compared to the average number of isoforms detected in the HBR_SIRV_Non datasets (62,277 isoforms). For the HBR_SIRV_Con libraries, an average of 33,457 of the detected isoforms were classified as Full-Splice_Match (FSM), indicative of isoforms that align perfectly to the annotated transcriptome reference. This results in a difference of 12,520 FSM isoforms over those identified from the HBR_SIRV_Non libraries (average of 20,937). Notably, SQANTI3 identified other isoform classes, the Novel_in_Catalog (NIC) and Novel_not_in_Catalog (NNC) which represent new, unannotated transcripts. The NIC isoforms contain novel combinations of known donor/acceptor splice sites, such as result from exon skipping, whereas the NNC isoforms contain at least one unannotated splice site. Here, the HBR_SIRV_Con read data yielded 1.3-fold more NIC and NNC combined isoforms compared to the HBR_SIRV_Non read data. Interestingly, the SQANTI3 classifier revealed that the HBR_SIRV_Con samples had an average of 14,999 isoforms that aligned outside annotated genes (i.e., intergenic), which was nearly six times higher than the non-concatenated samples. To resolve this observation more fully, we filtered the SQANTI3 outputs for only intergenic reads to better characterize the transcripts (Additional file 3). We found that the average intergenic isoform read length in the HBR_SIRV_Con samples was 1,233 bp, while in the HBR_SIRV_Non sample it was 3,475 bp; suggesting a preference for smaller isoforms to be preferentially concatenated thereby increasing overall intergenic reads and representation in the HBR_SIRV_Con samples.

**Fig. 4:**
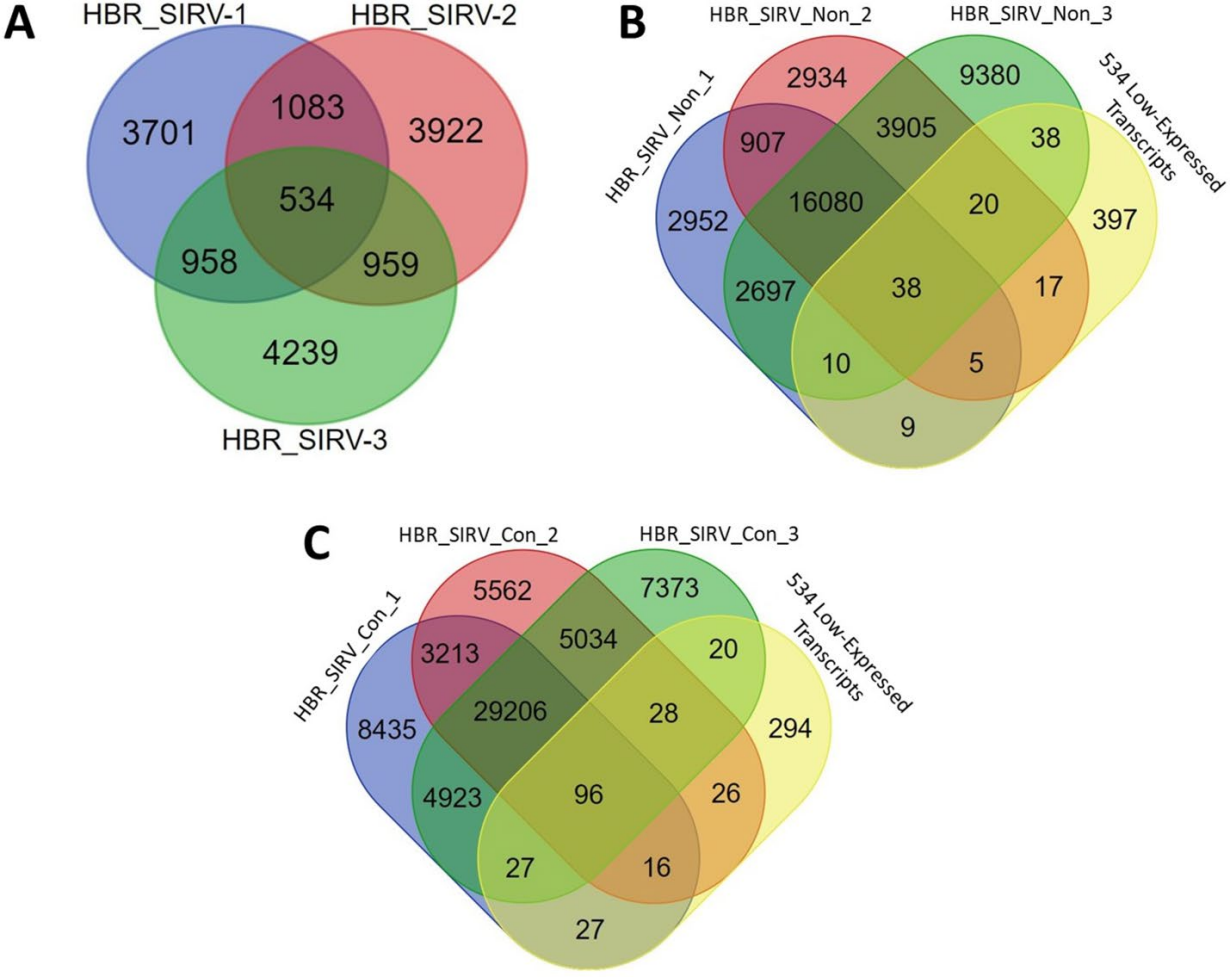
The number of detected low-level expressed transcripts in short and long-read HBR_SIRV RNA data. **A** Transcript per million (TPM) values were calculated from short-read RNA-Seq data for three replicates of the Human Brain Reference RNA mixed with Spike-In RNA Variant control molecules (HBR_SIRV-1, HBR_SIRV-2, HBR_SIRV-3). The figure A Venn diagram illustrates the number of transcripts with a TPM value equal to 0.5 (“low-expressed transcripts”) identified in each of the short-read HBR_SIRV sample replicates. In total, 534 low-expressed transcripts were identified that were shared between all three sample replicates. These 534 transcripts were used to assess low-level expressed transcript detection from long-read HBR_SIRV sample prep workflows. The Venn diagrams illustrate the number of lowly-expressed transcripts that were found in (**B**) long-read HBR_SIRV non-concatemer Iso-Seq sample datasets (HBR_SIRV_Non) or (**C**) long-read HBR_SIRV concatenated Iso-Seq sample datasets (HBR_SIRV_Con)

To determine whether the increased number of sequenced reads and detected isoforms with PB_FLIC-Seq led to improved identification of low-level expressed transcripts, we first identified lowly expressed transcripts in HBR_SIRV RNA using short-read RNA-Seq data and salmon analysis. Here, we considered an HBR_SIRV transcript as lowly expressed if the transcript per million (TPM) value was equal to 0.5, a TPM threshold in line with the Expression Atlas database’s standards for reporting RNA-Seq expression data [30]. Our examination of TPM values across three HBR_SIRV short-read RNA-Seq datasets (“HBR_SIRV-1, HBR_SIRV-2, HBR_SIRV-3”) revealed 534 transcripts meeting this criteria, each detected in every sample replicate (Fig. 4A, Additional file 4). Subsequently, we explored whether these 534 lowly expressed transcripts were present in the HBR_SIRV_Non and HBR_SIRV_Con long-read datasets, as assembled through the GFFCompare software tool (please refer to the methods section; Additional file 5). Among the 534 low-level expressed transcripts, 7.1% (38/534) were identified in all three HBR_SIRV_Non long-read datasets, with 25.7% (137/534) of these transcripts detected in at least one of the HBR_SIRV_Non replicates (Fig. 4B). As for the HBR_SIRV_Con samples, 18.0% (96/534) of the lowly expressed transcripts were detected in all three HBR_SIRV_Con replicates, while 44.9% (240/534) of these transcripts were found in at least one sample replicate (Fig. 4C).

**Fig. 5:**
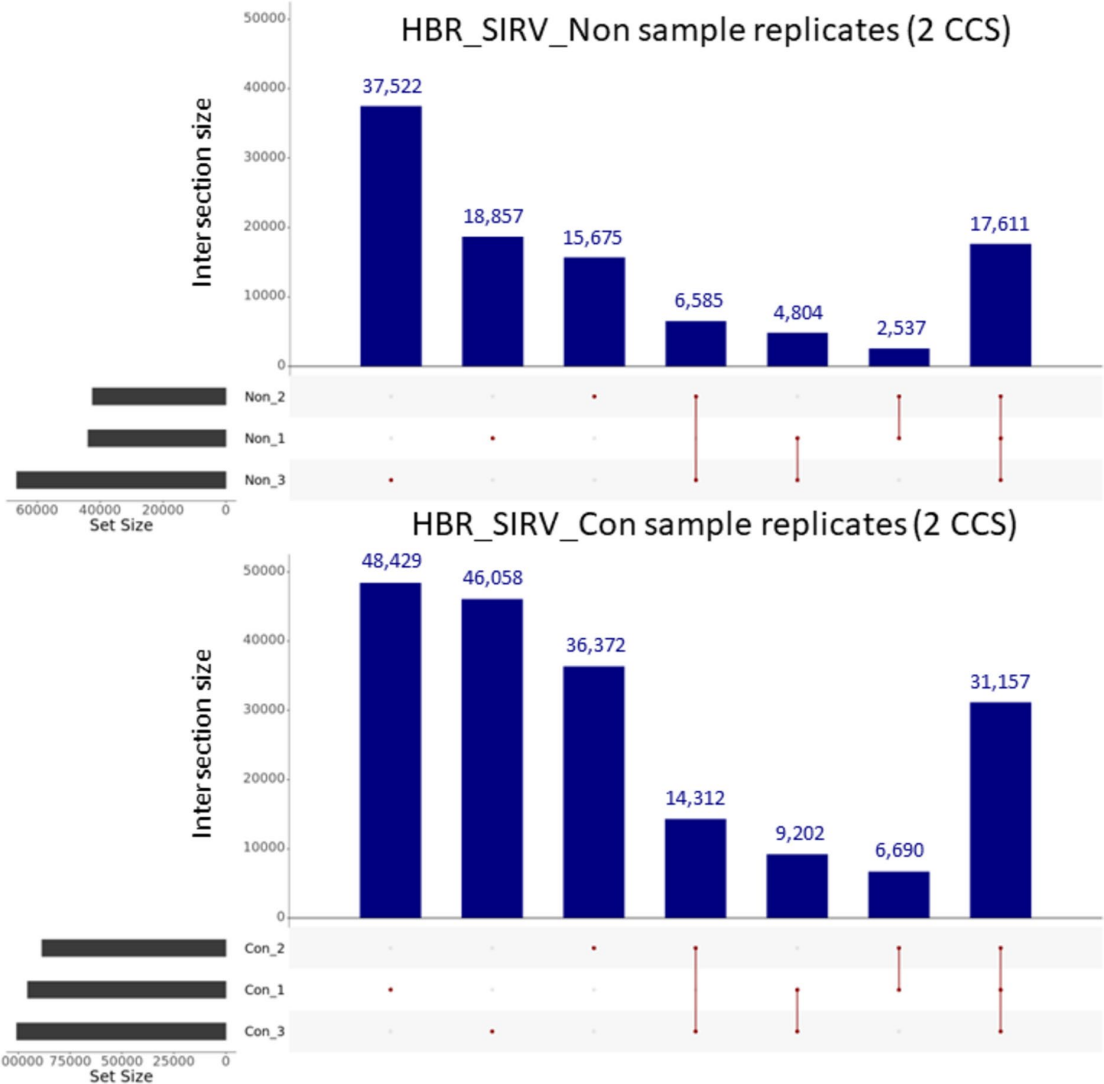
Detected isoforms supported by at least two Circular Consensus Sequencing “CCS” reads between sample prep methods. The samples include Human Brain Reference RNA mixed with Spike-In RNA Variants “SIRV” prepared with the PacBio Full-Length Isoform Concatemer Sequencing workflow “HBR_SIRV_Con” or prepared with a non-concatemer Iso-Seq workflow “HBR_SIRV_Non”. Upset plot illustrating the GFFCompare results for identifying unique isoforms among sample replicates. At least two CCS reads of support are required for isoform calling by the default PacBio Iso-Seq Analysis tool. Dots indicate isoforms unique to the sample, and a line connecting two or more dots indicate isoforms detected in multiple sample replicates. The numbers listed above the bar graphs represent the number of unique isoforms detected for that sample(s)

We evaluated the concordance of detected isoforms across sample replicates using the GFFCompare software tool [31] The GFFCompare tool takes all isoforms identified between sample replicates and generates a combined list of non-redundant transcripts and provides the number of reads supporting each transcript. This analysis revealed that the combined HBR_SIRV_Non replicates had a total of 103,591 unique isoforms (Fig. 5; Additional file 5), of which only 17.0% (17,611) were observed in all three sample replicates. The 103,591 unique isoforms were supported by a median depth of 3 CCS reads, and an average read depth of 18 CCS reads (Additional file 5). By comparison, GFFCompare output for the HBR_SIRV_Con data indicated a 1.9-fold increase in the number of unique isoforms (192,220) with a similar percentage of isoforms observed in all three sample replicates (16.2%; 31,157 isoforms). The 192,220 unique isoforms were supported by a median depth of 3 CCS reads, and an average read depth of 31 CCS reads.

**Fig. 6:**
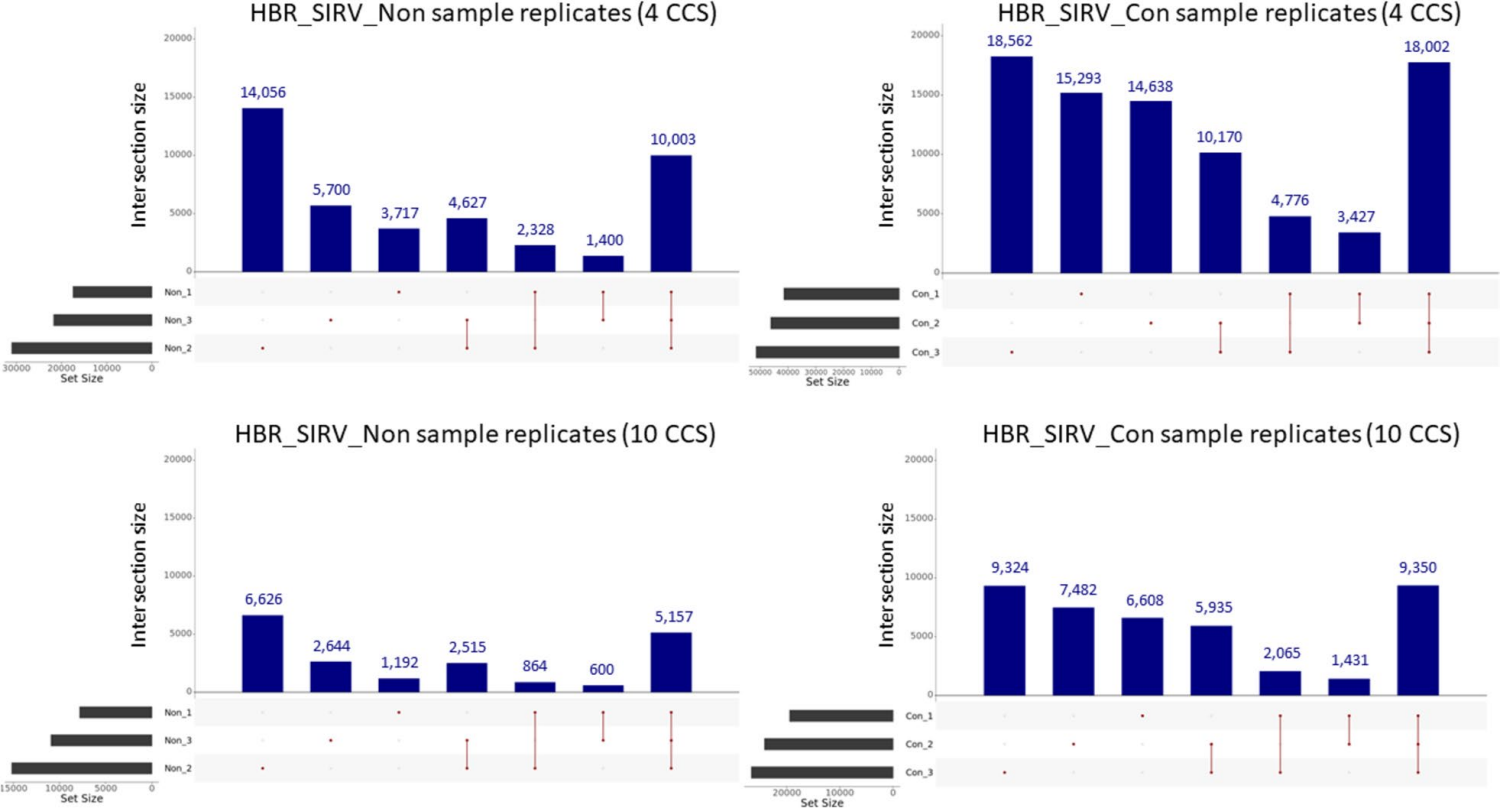
Detected isoforms supported by at least four or ten Circular Consensus Sequencing “CCS” reads between sample prep methods. The samples include Human Brain Reference RNA mixed with Spike-In RNA Variants “SIRV” prepared with the PacBio Full-Length Isoform Concatemer Sequencing workflow “HBR_SIRV_Con” or prepared with a non-concatemer Iso-Seq workflow “HBR_SIRV_Non”. Upset plot illustrating unique isoforms identified by GFFCompare analysis for the HBR_SIRV_Non and HBR_SIRV_Con sample set with required isoform read support of at least four or ten CCS reads. Dots indicate isoforms unique to the sample, and a line connecting two or more dots indicate isoforms detected in multiple sample replicates. The numbers listed above the bar graphs represent the number of unique isoforms detected for that sample(s)

Given that the default PacBio SMRT Link Iso-Seq Analysis tool necessitates a minimum of two CCS reads to support each isoform for it to meet filtering criteria, we raised the threshold to four and then to ten CCS reads. Our objective was to assess whether this adjustment could enhance the consistency of isoform detection across different samples and to establish appropriate read support thresholds for conducting differential isoform expression analysis across various sample types. To strike a balance between preserving a maximum number of unique isoforms and achieving improved consistency in the representation of isoforms across different samples, we set the four and ten CCS read thresholds. We based these cutoff values on the rationale that they are incrementally higher than the per-transcript median read support (three CCS reads) for both the HBR_SIRV_Con and HBR_SIRV_Non sample sets. The total number of unique isoforms passing filter for the combined HBR_SIRV_Non sample set decreased from 103,591 to 41,831, when requiring four or more supporting reads, with further reduction to 19,598 when requiring ten or greater supporting reads. As the required read support levels increased, the disparity with which an isoform was observed between sample replicates decreased. At the four CCS read threshold, 23.9% (10,003 of 41,831) of isoforms that pass read filtering are detected in all three replicates, with 43.9% (18,358) detected in at least two replicates (Fig. 6). A threshold of ten supporting reads further improved the overlap, with 26.3% (5,157 of 19,598) of isoforms being detected in all three replicates and 46.6% (9,136 of 19,598) detected in at least two replicates. The same filtering parameters were applied to the HBR_SIRV_Con sample set resulting in 84,868 and 42,195 total unique isoforms at the minimum four and ten supporting read filter, respectively. At these read support thresholds, the percentage of identical isoforms observed in all three sample replicates was 21.2% (18,002 of 84,868) and 22.2% (9,350 of 42,195). As expected, the percent isoform overlap improved when evaluating identical isoforms shared between at least two of the three sample replicates at 42.9% (36,375 of 84,868) and 44.5% (18,781 of 42,195). Thus, by performing the PB_FLIC-Seq workflow, we detected 2.2-fold more unique isoforms supported by at least ten CCS reads (42,195/19,598) compared to the same reference sample processed by the Iso-Seq workflow.

#### Evaluating PB_FLIC-Seq concatenation workflow: analysis of gene fusion artifacts

Since the PB_FLIC-Seq method involves the ligation-based concatenation of individual RNA transcript molecules, we sought to identify whether artifacts of this process could be detected as gene fusions. To evaluate the extent of fusion artifacts, we employed a two-step approach. In the first step, we examined the total number of fusions identified between the PB_FLIC-Seq workflow (HBR_SIRV_Con) and the non-concatemer Iso-Seq samples (HBR_SIRV_Non). We reasoned that significant increase in the overall number of fusion transcripts found within the HBR_SIRV_Con samples would suggest the possibility of PB_FLIC-Seq generating fusion artifacts. In the second step, we conducted an analysis of the gene fusion partners, specifically assessing whether any of them corresponded to the synthetic SIRV transcripts. This latter observation would indicate a potential issue with incomplete de-concatenation resulting in artificial fusion transcripts. To set a baseline level of total fusions identified and the gene partners involved, we processed the HBR_SIRV_Non data through our long-read fusion detection analytical pipeline, PB_FLIP, [17]. No fusion transcripts were predicted for one of the HBR_SIRV_Non samples, whereas the other two replicates had a total of seven unique fusion transcripts predicted (Additional file 6). By comparison, the PB_FLIP pipeline detected four, nine, and 11 fusion events representing a combined total of 13 unique fusion transcripts (Additional file 6). The variation in the total count of unique fusions detected in the HBR_SIRV_Con (13 fusion transcripts) and HBR_SIRV_Non samples (seven fusion transcripts) is more likely due to the substantial increase in both read counts (average of 3.4-fold increase) and detected isoforms (1.8-fold increase) in the HBR_SIRV_Con samples rather than stemming from incomplete de-concatenation leading to fusion artifacts. Next, we proceeded to assess the gene partners of the identified fusion transcripts. Of the seven unique fusion transcripts detected in the HBR_SIRV_Non data, two fusion transcripts (*AL445985.1*::*SPATA13* and *AC092691.1*::*LSAMP*) also were identified in at least one of the HBR_SIRV_Con samples. Notably, all four fusion transcripts identified in the HBR_SIRV_Con-1 sample also were identified in either the HBR_SIRV_Con-2 or Con-3 replicates. These fusion transcripts included *AC09269.1* (exon 1)::*LSAMP* (exon 2), *AC09269.1* (exon 1)::*LSAMP* (exon 6), *AC09269.1* (exon 1)::*LSAMP* (exon 8) and *RN7SK*::*MBP*. Importantly, none of the identified fusion transcripts for the HBR_SIRV_Con samples contained any of the synthetic SIRV isoforms as a gene partner.These findings collectively suggest a low likelihood of fusion artifact creation due to incomplete de-concatenation.

### Assessment of PB_FLIC-Seq workflow bias towards transcript length

**Fig. 7:**
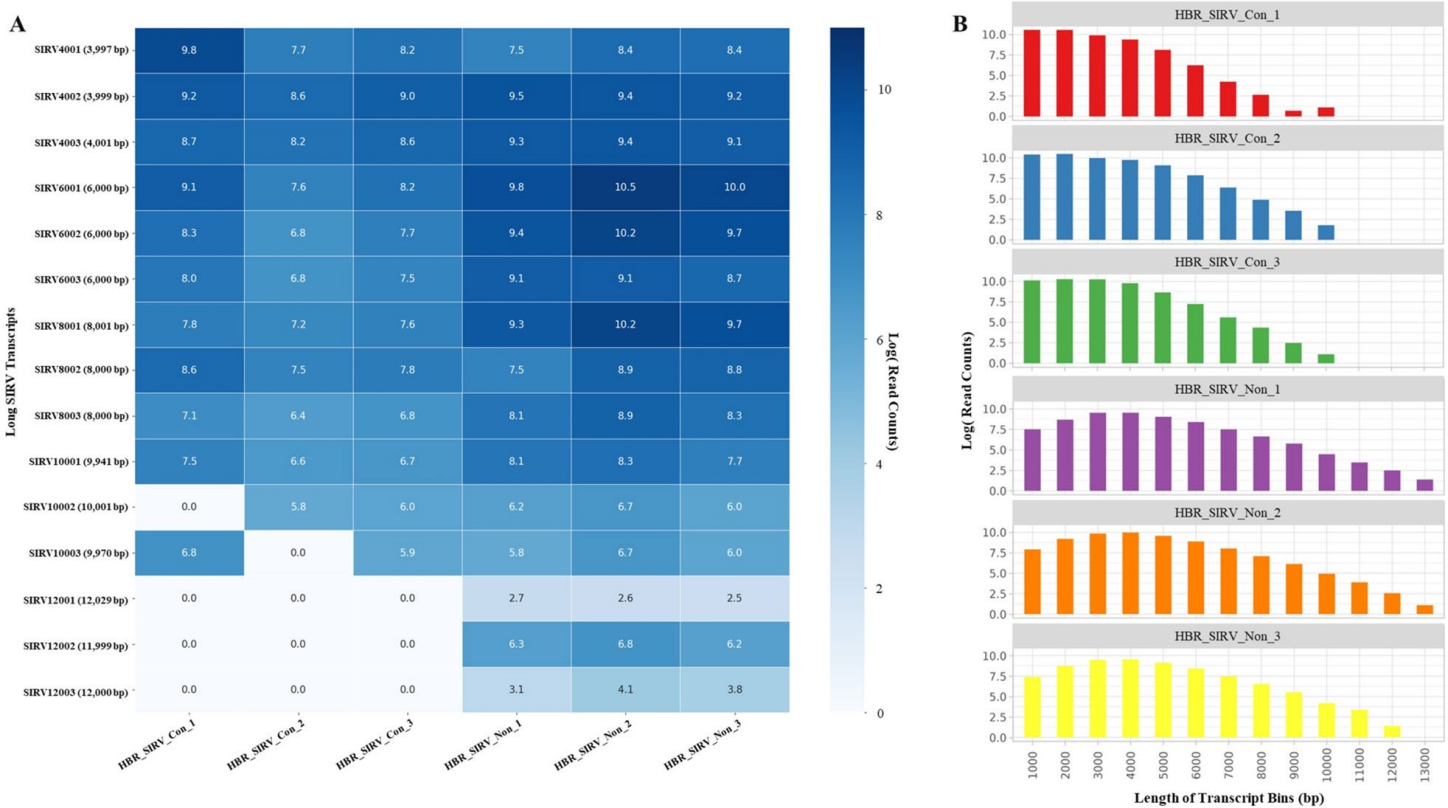
Long SIRV transcript recall and length assessment of detected transcripts between sample prep methods. The samples include Human Brain Reference RNA mixed with Spike-In RNA Variants “SIRV” prepared with the PacBio Full-Length Isoform Concatemer Sequencing workflow “HBR_SIRV_Con” or prepared with a non-concatemer Iso-Seq workflow “HBR_SIRV_Non”. **A** Heatmap illustrating detected full-length long SIRV transcript (y-axis) count among sample replicates (x-axis). The long SIRV transcript length is provided within the parenthesis next to SIRV transcript name on the y-axis. The long SIRV transcripts up to 10,001 bp (SIRV10001, SIRV10002, SIRV10003) are detected within the HBR_SIRV_Con samples, whereas all 15 long SIRV transcripts are observed in the HBR_SIRV_Non samples. **B** The bar plot illustrates the distribution of identified transcripts in the HBR_SIRV_Con and HBR_SIRV_Non samples, categorized into transcript length size-based “bins” with incremental 1,000 bp increments on the x-axis. The y-axis depicts the logarithmic representation of the read counts, indicating the level of support for transcripts within each respective bin

To assess if the PB_FLIC-Seq workflow is generating bias in observed transcript length, we first evaluated the long SIRV module that were spiked into the samples prior to library construction. The SIRV-Set 4 reference standard includes 15 equimolar RNA transcripts of roughly 4,000 (SIRV4001) to 12,000 (SIRV12001) bp not present in the human transcriptome thus providing a unique ability to interrogate concatenation bias in the context of isoform length resulting from the PB_FLIC-Seq workflow. The HBR_SIRV_Con libraries generated on average 37,860 full-length long SIRV reads, representing 12 unique long SIRV transcripts (Fig. 7A; Additional file 2). Notably, the 10,000 bp SIRV transcripts were detected in the HBR_SIRV_Con datasets with an average read support of 603, although no full-length reads were observed for the 12,000 bp SIRVs. All 15 long SIRV transcripts were identified in the HBR_SIRV_Non control libraries with an average total long SIRV read count of 109,355. Subsequently, to investigate potential transcript bias from a broader perspective, we categorized the identified transcripts from the SQANTI3 output based on their size for each sample and then aggregated the total read support for transcripts falling within these predetermined size ranges (Fig. 7B). Our analysis reveals a higher prevalence of shorter transcripts (1,000 – 3,000 bp) in the three HBR_SIRV_Con sample replicates when compared to the non-concatemer samples. Conversely, the non-concatemer samples exhibit a greater proportion of longer transcripts (> 6,000 bp). These findings collectively indicate a notable difference in transcript length between samples prepared using the PB_FLIC-Seq workflow and those prepared using Iso-Seq sample preparation.

### Utility of PB_FLIC-seq method to identify novel isoforms

We sought to illustrate the utility of the PB_FLIC-Seq workflow by studying RNA samples obtained from a single patient collected within an Institutional Review Board-approved translational research protocol of pediatric cancer patients. In particular, a 6-year-old male was diagnosed with diffuse midline glioma (DMG), a rare glial tumor of the central nervous system that is associated with poor outcomes. Short-read exome sequencing of the tumor revealed a somatic variant in the chromatin modifier gene *H3-3A* (NM_002107.4:c.83A > T:p.Lys28Met), which was subsequently clinically confirmed. Modifications in this H3 histone gene, particularly at the p.K28M residue, are known DMG oncohistone drivers and have been shown to cause DNA hypomethylation [32–34]. These changes can alter expression of the DNA repair enzyme, 06-methyl-guanine-DNA methyltransferase (*MGMT*), leading to poor prognoses [35, 36]. Given the association between H3 K28M variants and changes in gene expression, we prepared libraries from the cancer -and paired adjacent comparator tissue-derived RNAs using the PB_FLIC-Seq workflow. Subsequent PacBio sequencing and analysis were pursued to evaluate and compare the transcriptomics and identify any tissue-specific isoforms.

The s-reads for the tumor (9,714,94) and paired non-malignant adjacent tissue (10,547,041) were processed through our long-read analysis pipeline, PB_FLIP [17], for isoform characterization (Additional files 7 and 8). We initially assessed *MGMT* expression between the two tissue samples. *MGMT* expression may be epigenetically silenced by promoter hypermethylation in gliomas, [37] however prior clinical testing of this patient’s tumor sample confirmed that the *MGMT* promoter region was not hypermethylated. Our analysis of *MGMT* expression in the PB_FLIC-Seq data revealed four isoforms in the tumor and two isoforms in the comparator tissue. The four *MGMT* isoforms in the tumor were supported by a total of 199 reads, whereas the two *MGMT* isoforms in the comparator tissue were supported by a total of 134 reads.

In the setting of the translational research protocol, short-read RNA-Seq from diseased-involved tissue and, when accessible, adjacent non-tumor tissue comparator provides assessment of outlier gene expression [6] We combined short-read RNA-Seq expression data with the long-read PB_FLIC-Seq output from PB_FLIP to discern differential expression at isoform level resolution. To discern differential isoform expression, we adopted a two-step approach. In the initial step, we focused on evaluating the differential expression of annotated transcripts. This involved filtering the long-read SQANTI3 output to isolate Full-Splice_Match (FSM) isoforms in both the tumor and comparator tissue datasets, which served as the basis for our subsequent analysis. In the second step, we addressed novel isoform expression separately in both the tumor and comparator tissue samples by filtering the SQANTI3 output to extract only novel isoforms.

**Fig. 8:**
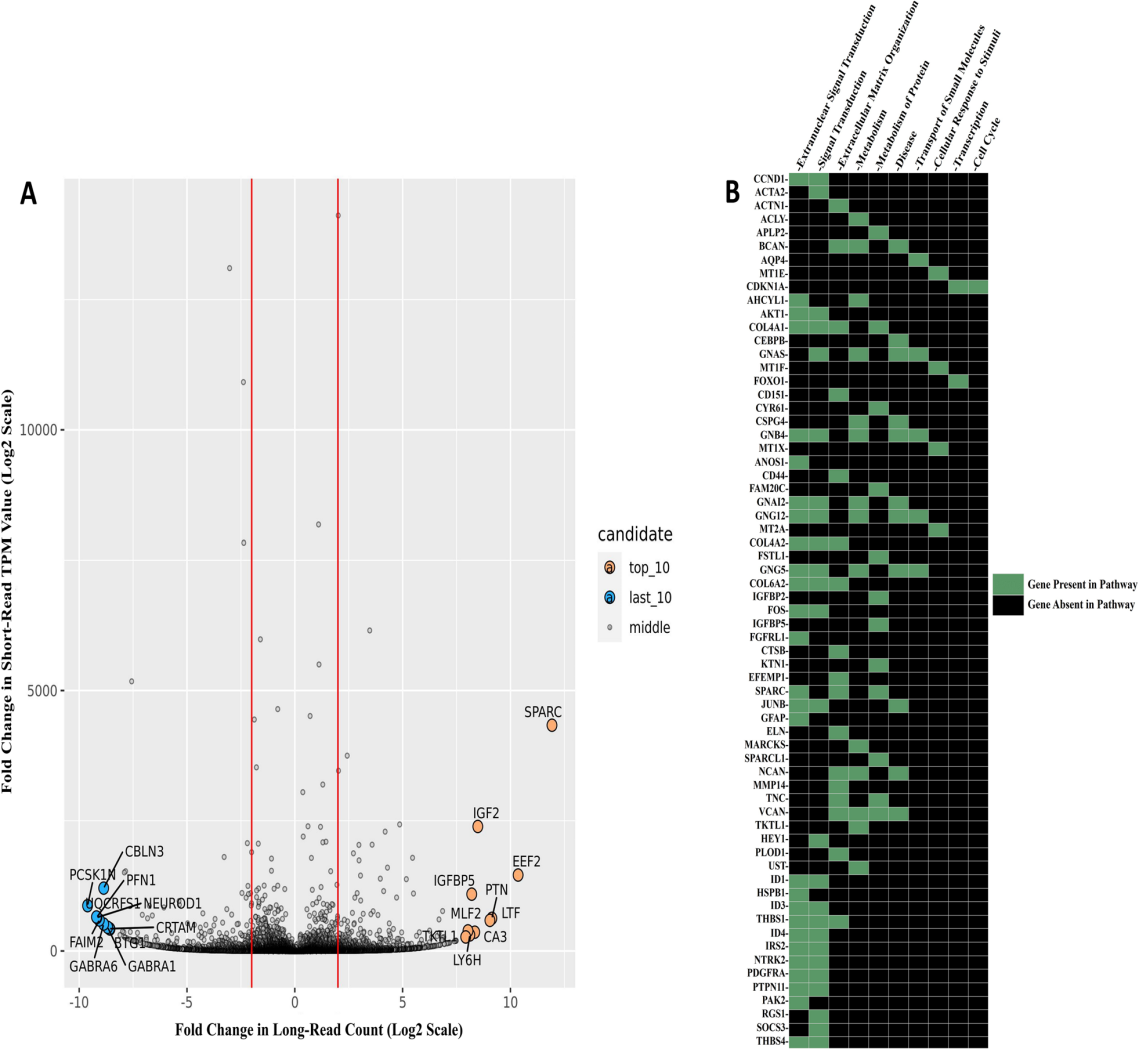
Expressed and upregulated genes detected in a pediatric diffuse midline glioma. **A** Correlation plot illustrating the fold-change in PacBio long-read count (x-axis) and fold-change in Illumina short-read transcript per million (TPM) value (y-axis) per isoforms identified in the tumor relative to the adjacent non-diseased tissue comparator sample. The genes represented in the correlation plot are the 1,799 transcripts that were found in the tumor sample with at least ten Circular Consensus Sequencing “CCS” long-reads of support and with short-read TPM value of ten or greater. **B** Heatmap of identified genes (y-axis) enriched in the tumor sample associated with their respective Reactome pathways (x-axis) wherein green indicates that gene is noted as being part of that pathway

In the first approach, we applied read support thresholds established from the HBR_SIRV datasets and identified 1,799 FSM isoforms in the tumor RNA sample supported by at least ten full-length long-reads from the PB_FLIC-Seq data that also have a short-read RNA-Seq determined transcript per million (TPM) expression value of ten or greater (Fig. 8A; Additional file 9). To discern isoforms preferentially expressed in the tumor sample, the 1,799 isoforms were further filtered to retain only those isoforms with at least five-fold higher long-read counts and short-read derived TPM values in the tumor relative to the adjacent tissue comparator RNA. Using this approach, only 262 isoforms remained after filtering, representing a total of 247 unique genes (Additional file 10); these 247 genes were uploaded into the Reactome database for pathway analysis.

Pathways identified from the Reactome analysis of the 247 genes enriched in the tumor sample, included processes involving immune system, signal transduction, metabolism, and extracellular matrix organization (Additional file 11). Clinical histopathologic review of this tumor reported an estimated 80% tumor cell content with microscopic analysis of tumor tissue showing evidence of necrosis, focal microvascular proliferation, and the presence of infiltrating macrophages. We therefore narrowed the pathway analysis results, excluding those genes involving the immune system, autophagy, and hemostasis to represent a more focused assessment of the disease process. Remaining pathways included extranuclear signal transduction with 26 unique genes identified in this pathway, signal transduction (25 genes), and extracellular matrix organization (17 genes) (Fig. 8B). Subsequently, we examined the transcript-level characteristics of these identified genes by utilizing data derived from the SQANTI3 isoform classifier. It is noteworthy that among these genes, 50% (32 of 64) express multiple FSM isoforms (Additional file 12).

The extracellular matrix (ECM) organization pathway was of particular interest as it included Secreted Protein Acidic and Cysteine Rich (*SPARC;* ENST00000231061.9), which had the greatest differential in long-read count (11,676-fold). ECM proteins have been reported to influence cell proliferation, adhesion, and migration [38] with additional studies suggesting that upregulation of *SPARC* could promote glioma invasion by altering the tumor microenvironment [39] We therefore further interrogated an additional 16 tumor-enriched ECM genes in our filtered list by evaluating the differential fold change in long-read count between tumor and adjacent comparator tissue sample. This assessment revealed seven ECM genes whose expressed isoform was in the overall top 50 enriched isoforms, including Collagen Type IV Alpha 1 and 2 Chain (*COL4A1*; ENST00000375820 and *COL4A2*; ENST00000360467), Collagen Type VI Alpha 2 Chain (*COL6A2*; ENST00000300527.8), Versican (*VCAN*; ENST00000513960), Thrombospondin 1 (*THBS1*; ENST00000260356), Actinin Alpha 1 (*ACTN1*; ENST00000193403), and Tenascin C (*TNC*; ENST00000350763). The expressed ECM isoforms of these eight total genes are supported by an average full-length long-read count of 1,704 (range of 92 to 11,676) compared to an average of one (range of zero to six) full-length read count in the adjacent comparator tissue. In further validation of this differential expression, the average short-read TPM value of these expressed isoforms was 45 in the tumor sample and three in the comparator tissue, a 15-fold increase, suggesting an elevated ECM pathway.

To complement our analysis on differential isoform expression, we next evaluated novel isoforms of annotated genes that were uniquely detected in the tumor sample. Here, only novel isoforms that contained at least ten long-reads of support in the tumor and were undetected in the non-malignant tissue comparator were evaluated (Additional file 13). Furthermore, it’s important to note that the assessment of novel isoform expression did not involve the utilization of short-read RNA-Seq TPM values. This is because unannotated isoforms are not included in the reference transcriptome, resulting in the absence of any assigned TPM values. The highest expressed novel isoform was from SPARC-related Modular Calcium-Binding Protein (*SMOC1*), a gene in the SPARC matricellular protein family. While elevated *SMOC1* expression and protein levels in brain cancer compared to normal brain, was previously reported, [40] differential *SMOC1* isoform expression has not. Here, we noted a novel, tumor-unique *SMOC1* isoform identified as Novel_in_Catalog by the SQANTI3 classifier supported by 1,038 long-reads. After manual interpretation of isoforms aligned to this locus using the Integrative Genomics Viewer, [41] this novel isoform demonstrated skipping of exons six and seven of the canonical transcript (ENS00000361956.7) (Supplemental Fig. 1A). The exon skipping results in an in-frame loss of amino acid residues 176 – 222, a region of polar residues, that are not further annotated with respect to function. To substantiate the discovery of the novel *SMOC1* isoform through long-read sequencing, we aligned short-read RNA-Seq data and generated a Sashimi plot, revealing evidence of the same exon skipping (see Supplemental Fig. 1B). However, it's important to note that, unlike long-read sequencing, the accuracy of short-read assembly would determine the resolution of the full-length transcript.

In addition to the novel *SMOC1* isoform, a novel isoform of the Cellular Communication Network Factor 2 (*CCN2*) gene, a matricellular protein that associates with the cell surface and ECM [42], was identified as a Novel_in_Catalog isoform. The *CCN2* isoform was supported by 269 long-reads and retained introns two and three of the canonical transcript (ENST00000367976.4; 349 amino acids) (Supplemental Fig. 2A). Through the retention of intron two, a stop codon is introduced. The distance from the end of exon two to the beginning of exon four of *CCN2* (chr6:131,195,770 to chr6:131,195,160) exceeds the detection limit from short-read sequencing platforms. Therefore, phasing of the two *CCN2* intron retention events from short-read data to determine whether the retained introns are within the same transcript and thus result in an in-frame full-length transcript would not be possible. In addition, short-read RNA-Seq analysis tools cannot reliably detect intron retention events [43]. To ascertain the detectability of the novel *CCN2* isoform using short-read RNA-Seq data, we created a Sashimi plot from short-read RNA-Seq (Supplemental Fig. 2B). This plot indicated that neither *CCN2* intron retention was easily detectable from short-read RNA-Seq data alone. Here, the long-read Iso-Seq data fully resolves the two intron retention events revealing both occur within the same *CCN2* isoform, and predict a truncated protein. While altered expression of *CCN2* has been associated with tumor survival and progression, [44] it is unclear how or whether this predicted protein product would influence disease biology.

## Discussion

RNA-Seq provides an unbiased study of the transcriptome and has been used in many aspects of cancer research including defining tumor heterogeneity and immune microenvironment using single cell transcriptome profiling, [45, 46] discovery of biomarkers and cancer neoantigens, [47] and identification of tumor-specific differentially expressed genes [48]. Furthermore, RNA-Seq enables transcriptome profiling at the isoform level, revealing novel isoforms that may be associated with disease [49] or differential development across normal tissues. Thus, correctly identifying sample-specific isoforms can inform biology. To identify known and novel isoforms, we developed a long-read RNA-Seq workflow that inherently overcomes challenges in short-read sequencing for isoform discovery, [15, 50] while optimizing the sequencing capacity and breadth of transcript expression level explored by long-read sequencing. Our approach, PB_FLIC-Seq, leverages a transcript concatenation strategy to enhance the number of sequenced reads on the PacBio platform. This strategy is made feasible primarily due to two key factors. First, the PacBio platform boasts the capability to sequence molecules up to 15,000 bp in length while maintaining a high read accuracy of 99.9% [24]. Second, is the fact that the average length of expressed transcripts is well below the 15,000 bp threshold. This is evident in the length distribution plot (Fig. 7B) and aligns with the reported average human transcript length of 1,600 ± 1,100 bp [27]. The significant differential between expressed transcript length and the sequencing capacity of the PacBio platform enables us to potentially concatenate a significant percentage expressed genes. Using a commercial reference standard RNA mixture containing known isoforms through our concatenation workflow, PB_FLIC-Seq, we demonstrated a 3.4-fold increase in the number of long-reads sequenced over the same control samples processed with Iso-Seq, a non-concatemer approach. The gain in output reads resulted in a 1.8-fold increase in the total number of unique isoforms detected, with novel isoforms observed at a 1.3-fold higher abundance compared to controls. To this end, the PB_FLIC-Seq workflow can be used to more fully resolve the expressed isoform representation within a given sample. Such resolution is needed to accurately interrogate the isoform landscape between matched sample types, such as a tumor and adjacent non-diseased tissue comparator.

In one such example, we applied the PB_FLIC-Seq workflow to a patient-derived cancer and adjacent non-malignant tissue sample, assessing tissue-specific isoform expression. Data analysis and comparison identified novel and differentially expressed tumor-specific isoforms. While not required to initiate analysis within PB_FLIC-Seq, generated short-read RNA-Seq data from the same tissues provided an orthogonal method to support the validity of the long-read findings. We included Reactome database pathway analysis for gene isoforms of interest to provide a pathway filtering mechanism that identified highly-expressed, tumor-unique isoforms of genes having a known association with the ECM. Upregulation of ECM pathways have been reported in a prior differential expression study that profiled a cohort of 35 pediatric diffuse intrinsic pontine glioma (DIPG) and ten normal brain samples [51]. Another study showed alteration in the ECM space correlates with promotion of malignant tumor cells, [52] suggesting ECM components as potential new targets for emerging therapeutic approaches. In further support of this therapeutic conclusion, overexpression of genes involved in modulating ECM constituents have been reported in glioma cells with a demonstrated role in diminishing efficacy of other cancer therapeutics [53].

In this patient, the presence of the novel *SMOC1* and *CCN2* isoform in the DMG tumor tissue, while being absent from adjacent tissue, underscores the fact that the tumor transcriptome represents a complex interplay of alternative and aberrant splicing potentially impacting tumorigenesis in ways that are not yet fully understood.

While our PB_FLIC-Seq method allows for increased depth and ability to detect isoforms, we identified two limitations where continued efforts and new offerings from industry would further improve the demonstrated benefits of the method. One area for development was the loss of the long SIRV spike-in transcripts that were greater than 10,000 bp in size. In the current iteration, our protocol uses streptavidin bead-based capture to remove template switching oligo (TSO) priming artifacts that occur during reverse transcription. While strategic selection of streptavidin beads was considered for large molecule binding efficiency, capture performance for molecules greater than 2,000 bp is significantly reduced because of steric hindrance, likely explaining the loss in detecting the long SIRV transcripts. Another challenge of employing Iso-Seq for isoform characterization is that the current Iso-Seq cDNA generation process does not include the use of unique molecular identifiers to discern PCR duplicates from true biological expression. However, efforts to include unique molecular identifiers within the Iso-Seq protocol have been described [54]. With improvements such as depletion-based strategies of unwanted overexpressed transcripts, [55] concatenation workflows like PB_FLIC-Seq, and sequencing platform upgrades such as the Revio that offer larger SMRT Cell capacity, [56] Iso-Seq provides a new area of investigation focusing on alternative splicing and isoform differences in comparator and disease tissues that may control phenotypic presentation.

## Conclusion

We present a concatenation-based method (PB_FLIC-Seq) for increasing the number of full-length RNA sequencing reads generated on the PacBio platform. By using a commercial reference RNA and our methodology, we demonstrated an increase in the total number of reads (3.4-fold higher), resulting in an increase in overall number of unique isoforms identified (1.8-fold higher) when compared to a non-concatemer method. The PB_FLIC-Seq workflow was applied to a pediatric DMG and adjacent non-malignant tissue comparator, revealing tumor-specific novel isoform expression. Such methods can improve the accurate characterization of transcript isoforms in cancers and other disease processes, as well as in normal tissues.

## Methods

### RNA samples and description

This study used the Human Brain Reference (HBR) RNA (Ambion Cat. No. AM7962, Austin, TX) spiked with a 2% SIRV-Set 4 synthetic RNA mix (Lexogen Cat. No 141, Vienna, Austria). Within the Lexogen Spike-In RNA Variant (SIRV-Set 4) user guide, the mRNA content for HBR is noted as 2% of total HBR RNA. Therefore, to achieve a 2% SIRV spike-in within the final library, 120 pg of SIRV-Set 4 was added to 300 ng total HBR RNA for library preparation. The HBR RNA sample mixed with 2% SIRV-Set 4 will be abbreviated as HBR_SIRV for the remainder of the text.

The SIRV-Set 4 module is a set of synthetic transcripts that provide processing controls for determining isoform complexity based on length, alternative splicing events, and abundance. The SIRV-Set 4 module includes long SIRV transcripts, SIRV isoforms, and the External RNA Control Consortium transcripts. The long SIRV module is comprised of 15 total RNA transcripts, with three transcripts each at the following sizes of roughly 4,000, 6,000, 8,000, 10,000, and 12,000 bp. The SIRV-Set 4 isoform module includes seven genes representing 69 isoforms with lengths ranging from 160 to 2,940 bp. These isoforms represent a mix of mono- and multi-exonic transcripts, alternative start/stop sites, and antisense transcripts, thus providing a unique synthetic control to account for isoform complexity.

In addition, this study included total RNA extracted from a pediatric diffuse midline glioma and comparator adjacent tissue. The pediatric diffuse midline glioma and comparator tissue total RNA was DNase treated and size-selected using the Zymo RNA Clean & Concentrator-5 Kit (# R1014) and used as input into library prep.

### PacBio Iso-Seq cDNA concatenation sample prep and Sequel IIe sequencing

For each sample, setup of first-strand cDNA synthesis occurred in duplicate PCR tubes each containing 300 ng of total tumor, comparator adjacent tissue or HBR_SIRV RNA. The first-strand cDNA synthesis protocol uses oligo(dT) priming of polyadenylated mRNA as outlined within Iso-Seq Express Template Procedure & Checklist (PN 102–396-000 (APR2022)) using the NEBNext Single Cell/Low Input (Cat. No E6421S, Ipswich, MA) kit. A template switching mechanism is used to generate full-length first-strand cDNA. During reverse transcription, terminal transferase activity provides a non-templated dCTP anchor. The anchor allows the annealing of the Iso-Seq express template switching oligo (TSO), incorporating the oligo sequence into the first-strand cDNA. Synthesized first-strand cDNA was purified using 1.3 × SMRTbell beads with final elution in 40 µl of Buffer EB (Qiagen, Hilden, Germany).

The first-strand cDNA synthesis reaction can result in off-target TSO priming artifacts. Therefore, a streptavidin-bead based capture of PCR amplified biotinylated cDNA product was used to remove TSO priming artifacts. During first-strand synthesis, the addition of the 5' TSO and 3' oligo(dT) site allows for full-length cDNA amplification [57]. The TSO deletion PCR amplification used the following reaction conditions: 50 µl Kapa HiFi Uracil + Ready Mix (2x) (Roche # 7,959,079,001), 5 µl of primer Iso-Seq_Concat_TSO (10 µM, IDT), 5 µl of primer Non-poly(dT) (10 µM, IDT), and 40 µl of purified first-strand cDNA. The 3’ non-poly(dT) primer was modified to include a 5’ biotin tag followed by deoxy-uracil nucleotides (Additional file 14). PCR cycling conditions were the following: 98 °C for 3 min, followed by 15 cycles of 98 °C for 20 s, 65 °C for 30 s and 72 °C for 10 min with final 72 °C extension for 10 min. Post-PCR reactions were purified using 1.3 × SMRTbell beads with final elution in 40 µl of Buffer EB. The PCR product was quantified using Qubit (Thermo-Fisher # Q32851) with size distribution evaluated using the Bioanalyzer High Sensitivity DNA kit (Agilent # 5067–4626). Purified PCR product (range of 288 – 1,100 ng) was mixed with 10 µL (100 µg) Dynabeads™ kilobaseBINDER™ (Thermo-Fisher #60,101) to remove cDNA derived from TSO priming after binding and washing steps. Final bead reconstitution used 41µL TE (Thermo #AM9849), followed by addition of 2 µL USER (Uracil-Specific Excision Reagent) Enzyme (NEB #M5505S, which creates single nucleotide gap at each uracil residue), with incubation at 37 °C for 2 h to uncouple the bound cDNA molecules from the beads. Following USER digestion, the reaction was placed on a magnet for 5 min to separate the beads from unbound cDNA in the supernatant. The supernatant from duplicate reactions, that belong to the same sample source, were combined for subsequent purification and cDNA size-selection.

Purified cDNA was size-selected by enriching molecules > 2,000 bp using a 0.4 × SPRIselect paramagnetic bead solution (Beckman Coulter, Brea, CA). The resulting supernatant containing unbound cDNA was mixed with additional SPRIselect beads for a final 0.6 × ratio yielding a double-sided size-selected product (0.4/0.6x) between 500 – 2,000 bp. The two fractions of size-selected cDNA were eluted in 20 µL EB buffer and used as input into the bulk Iso-Seq concatemer PCR reaction. The 0.4 × SPRIselect size-selected cDNA was distributed into 2 PCR tubes (10 µL of cDNA per tube; range of 5.6 – 37.8 ng total mass) with each PCR tube containing: 50 µl Kapa HiFi Uracil + Ready Mix (2x) (Roche # 7,959,079,001), 10 µl of 5 µM primer mix (Additional file 14), and 30 µl of nuclease-free water. The 0.4/0.6 × SPRIselect size-selected cDNA was distributed into 4 PCR tubes (2.5 µL of cDNA per tube; range of 11.4 – 37.2 ng total mass) with each PCR tube containing: 50 µl Kapa HiFi Uracil + Ready Mix (2x) (Roche # 7,959,079,001) containing dNTPs and polymerase, 10 µl of 5 µM primer mix (Additional file 14), and 37.5 µl of nuclease-free water. PCR cycling conditions were the following: 98 °C for 3 min, 8 cycles of 98 °C for 20 s, 65 °C for 30 s and 72 °C for 10 min; final 72 °C extension for 10 min. Post-PCR reactions were purified using 0.7 × SPRIselect beads with final elution being 30 µl in Buffer TE quantified using Qubit (Thermo # Q32851) and size distribution evaluated with Bioanalyzer High Sensitivity DNA Kit (Agilent # 5067–4626). In a subsequent reaction, 500 ng of each 0.4 × SPRIselect PCR product in 58 µL TE buffer was added to 2 µL of USER® Enzyme (M5505S) and set to incubate at 37 °C for 2 h. In a separate reaction, 300 ng each of the 0.4/0.6 × PCR products in 58 µL TE buffer was added to 2 µL of USER® Enzyme (M5505S) and set to incubate at 37 °C for 2 h. Following USER digestion, 2 µL of HiFi Taq DNA Ligase (M0647S) and 6.8 µL of HiFi Taq DNA Ligase buffer was added to each reaction and incubated in a thermocycler at 42 °C for 2 h. After ligation, the reaction was purified using 1.3 × SMRTbell clean up beads, eluted in 25 µL EB buffer and quantified using Qubit (Thermo # Q32851) and Genomic DNA ScreenTape (Agilent #5067–5365).

SMRTbell library preparation of the concatenated cDNA fractions used the PacBio SMRTbell Express Template Prep Kit 3.0 protocol (PN 102–166-600 (Apr 2022); Menlo Park, CA) with the following modifications. Ligation reaction used barcoded SMRTbell adapters with incubation overnight for 16 h at 20 °C. The post-ligation reaction was mixed with 1.0 × SMRTbell cleanup beads using gentle rotation on a hula mixer at 9 rpm for 20 min at room temperature. Final elution occurred in 40 µL EB buffer with purified SMRTbell library used as input into a nuclease treatment step. Post-nuclease treatment, the concatemer SMRTbell libraries were size-selected for molecules > 1,000 bp using 0.5 × Ampure PB bead size selection kit (PacBio # 102–182-500) with final elution in 20 µL EB buffer.

SMRTbell library molarity was calculated using concentration and sized based on Qubit dsDNA HS assay readings (Thermo Fisher Scientific, Waltham, MA) and the Genomic DNA ScreenTape (Agilent # 5056–5365) for size distribution. For each sample, the 0.4 × SPRIselect cDNA concatemer (dimer) and the 0.4/0.6 × SPRIselect cDNA concatemer (tetramer) were pooled at an equimolar ratio into a single reaction for SMRTbell complexing. SMRTbell libraries were complexed following the SMRTLink Sample Setup for HiFi library instructions, with Sequel II Binding Kit 3.2 and sequenced at a 60 pM on-plate loading concentration. Sequencing was performed on the PacBio Sequel IIe platform using 8 M SMRT Cells (PacBio # 101–389-101) and Sequel II Sequencing Kit 2.0 (PacBio # 101–820-200). Sequencing included a 2h pre-extension and a 30h movie collection. The HBR_SIRV was prepared using the described protocol in triplicate reactions for assessment of reproducibility and to generate analytical statics.

### PacBio SMRT link data processing

Primary data was analyzed with PacBio SMRT Link v.11.1.0.166339, [58] generating demultiplexed circular consensus sequence (CCS) reports and output files required for use in the following SKERA application [59]. SKERA was used in split mode with default parameters to trim MAS-Seq adapters (Additional file 15) and to deconcatenate reads into individual segmented reads (s-reads). The s-reads were processed using lima [60] which removes Iso-Seq specific primers that were added during cDNA synthesis to generate full-length reads. The lima software was run in specialized –*isoseq* mode and the parameter –*peek-guess* to accommodate more than one pair of 5’ to 3’ primer sequences. The resulting full-length reads were then used as input into the IsoSeq3 refine application [61] to trim the poly(A) tail and to remove any remaining concatemer reads. Reads that remain are classified as full-length non-concatemer (FLNC) reads. Next, the IsoSeq3 cluster tool [61] collapses two or more FLNC reads that differ by < 100 bp on the 5’ end, and < 30 bp on the 3’ end, and has no internal gaps that exceeds ten bp. The resulting collapsed FLNC are defined as High Quality Isoforms (HQ_Isoforms).

### Isoform characterization by PB_FLIP

The HQ_Isoforms were processed using the PacBio Long-Read Fusion and Isoform pipeline (PB_FLIP) for isoform characterization and fusion calling using previously defined analysis parameters [17]. Here, HQ_Isoforms are classified using the SQANTI3 analysis tool [62] which categorizes isoforms into defined classes based on splice junctions. As previously described, these classes categorize isoforms as both novel and known [29]. Step 4 of PB_FLIP isoform analysis provides a list of defined SQANTI3 full-length detected isoforms, including those isoforms from the known SIRV spike-in. The full-length SIRV isoform read counts were manually curated and used as input for a seaborn package-based, in-house python script that generates heat maps to illustrate differential detection between the standard and PB_FLIC-Seq workflow. For fusion detection, the PB_FLIP analysis tool requires a HQ_Isoforms to map to two or more separate loci, a minimum of 5% of the read must align to each locus, alignment coverage of combined loci must be at least 99%, and the fusion must be supported by a minimum of five FLNC reads for that fusion event to pass filtering parameters.

### GFFCompare analysis for sample-to-sample isoform comparison

Detected HQ_Isoforms were compared between samples using a GFFCompare software (v.0.12.6) tool [31]. In this software and using default parameters, except for the removing of those isoforms marked as “unknown” (GFFCompare class code -u), transcriptome features captured within sample specific General Feature Format (GFF) files are compared to a provided GRCh38 reference generating a combined list of non-redundant isoforms (reference annotation GFF file) observed across the multiple samples. The combined reference annotation GFF file is then used as a comparator to each individual sample GFF file to identify presence or absence of isoforms detected for that given sample. By default, the PacBio SMRT Link Iso-Seq Analysis applications requires at least two CCS reads of support for a HQ_Isoform to pass filtering parameters. We evaluated this read threshold using the GFFCompare output with “unknown” isoforms removed and then by changing the HQ_Isoform read support cutoff to four and ten CCS reads of support within the PB_FLIP Isoform Step 2 output file. RStudio was used to create upset plots that were generated from the number of unique isoforms identified by the GFFCompare software tool for each sample replicate at the various read support thresholds.

### Reactome pathway analysis for the pediatric diffuse midline glioma sample

An in-house R script was used to merge the output files generated from step 4 of PB_FLIP isoform characterization (file squant3_canonical_result.tsv) for both the tumor and the paired adjacent tissue sample containing gene name, associated transcript, short-read transcript per million (TPM) expression values and the number of long-read read counts for each isoform detected. Next, the list of detected tumor isoforms was manually filtered to retain only those isoforms supported by at least ten full-length long-read counts from the Iso-Seq data that also has a short-read TPM expression value of ten or greater. For remaining isoforms, to determine which of the remaining expressed isoforms were enriched in the tumor sample, differential fold-change for long-read count and short-read TPM value was calculated for each isoform between the tumor and adjacent tissue sample. For calculating fold-change, those isoforms with zero reads of support had an assumed value of one. Enriched isoforms in the tumor were classified as those with five-fold more long-read counts and at least five-fold higher short-read derived TPM values in the tumor over adjacent tissue RNA sample (Additional file 10). The Over-Representation Analysis (ORA) was performed using Reactome (v.84) web-based portal [63] and the curated list of 247 enriched genes identified in the pediatric diffuse midline glioma sample to evaluate Reactome pathways that are over-represented in the gene list. Then significant pathways were identified by setting a false discovery rate (FDR) greater than or equal to 0.5 (Additional file 11).

### Vignette patient material

Short-read RNA-Seq sample prep included DNase treatment (Zymo Research #R1081) of total tumor and adjacent non-malignant tissue RNA (1,000 ng). The resulting RNA was ribodepleted using the Ribo-Zero Complete Globin kit with library construction using the TruSeq Stranded Total RNA kit (Illumina #20,020,612). Paired-end 151-bp reads were generated on Illumina HiSeq 4000 and aligned to the human genome reference sequence build GRCh38 using a custom in-house pipeline and the splice-aware aligner STAR [64]. Salmon version 0.13.1 was used to calculate TPM values as previously described [17]. In addition, the tumor was previously characterized via exome sequencing and found to have a mutation in *H3-3A* (NM_002107.4:c.83A > T:p.Lys28Met) [65]. The *H3-3A* K28M mutation had been clinically confirmed, likely via sanger sequencing, at the patient’s prior institution but we do not have access to those records. In addition, a clinical assay of MGMT promoter methylation was also performed on the tumor at an outside institution.

### Short-read RNA-Seq sample prep and sequencing for HBR_SIRV RNA

Triplicate reactions of the HBR_SIRV RNA (500 ng input) was prepared using the NEBNext rRNA Depletion Kit v2 (New England Biolabs #E7400S) for ribodepletion and the NEBNext Ultra II Directional RNA Library Prep Kit for Illumina (New England Biolabs #E7760S) for library construction. Paired-end 151-bp reads were generated on Illumina NovaSeq 6000 and aligned to the human genome reference sequence build GRCh38 using a custom in-house pipeline and the splice-aware aligner STAR [64]. Salmon version 0.13.1 was used to calculate TPM values as previously described [17]. The Sashimi plots were made using ggsashimi.py script [66] for CCN2: chr6:131,948,176–131,951,372, and SMOC1:chr14:69,954,131–70,032,366 with read cutoff set to 10.

### Supplementary Information

**Additional file 1. **Summary of PacBio HiFi and SKERA read metrics.

**Additional file 2. **Read counts for the SIRV isoform and long SIRV transcript detected in HBR_SIRV_Con and HBR_SIRV_Non samples.

**Additional file 3. **SQANTI3 intergenic isoform read lengths.

**Additional file 4. **Lowly-expressed transcripts characterized as those transcripts with a TPM value equal to 0.5 identified in three replicates of HBR_SIRV short-read RNA-Seq.

**Additional file 5. **GFFCompare output illustrating unique isoforms detected and read support for the HBR_SIRV_Con and HBR_SIRV_Non samples.

**Additional file 6. **Gene fusions called passing filter by PB_FLIP analysis. 

**Additional file 7. **Tumor SQANTI3 output for isoform characterization.

**Additional file 8. **Adjacent non-malignant tissue SQANTI3 output for isoform characterization.

**Additional file 9. **Isoforms identified in the tumor sample with a short-read TPM value greater than 10 and a long-read support of 10 or greater.

**Additional file 10. **Tumor enriched genes with long-read count and short-read TPM value 10 or greater. 

**Additional file 11. **Reactome pathway analysis for the 247 tumor enriched genes, filtered for FDR < = 0.05.

**Additional file 12. **Transcript level characterization of enriched genes in the tumor.

**Additional file 13. **Long-read SQANTI3 classified novel isoforms enriched in the pediatric diffuse midline glioma.

**Additional file 14. **List of primers used in the PB_FLIC-Seq workflow.

**Additional file 15. **List of PB_FLIC-Seq barcodes used for SKERA deconcatenation.  

**Additional file 16: Supplemental Figure 1. **(A) Illustration of the tumor-specific novel SMOC1 isoform detected from the long-read PB_FLIC-Seq workflow showing exon skipping of exons 6 and 7 (highlighted by red box). (B) Sashimi plot generated from tumor and adjacent tissue comparator short-read RNA-Seq data showing exon skipping (highlighted by red box) within tumor SMOC1 expressed transcripts. **Supplemental Figure 2.** (A) Illustration of the tumor-specific novel CCN2 isoform detected from the long-read PB_FLIC-Seq workflow showing intron retention of introns 2 and 3 (highlighted by light orange box). (B) Sashimi plot generated from tumor and adjacent tissue comparator short-read RNA-Seq data showing splicing events (red box highlights introns of interests) for expressed CCN2 transcripts.

## Data Availability

The PacBio long-read RNA sequencing (Iso-Seq) data presented in this publication for HBR_SIRV is pending upload to the NCBI database of Sequence Read Archive (SRA) under submission number SUB13982783. Illumina short-read RNA-Seq data for the diffuse midline glioma has been deposited in the NCBI dbGaP and is available through accession number phs001820.v2.p1 under sample IGMJL17012. The PacBio long-read data for the diffuse midline glioma and adjacent non-malignant comparator tissue sample is pending upload to the dbGaP accession number phs001820.v3.p1.
