# Supplementary material for: Full-length isoform concatenation sequencing to resolve cancer transcriptome complexity
Source: BMC Genomics. 2024 Jan 29;25:122. doi: 10.1186/s12864-024-10021-x (PMC10823626; doi:10.1186/s12864-024-10021-x)
Supplement: Supplementary file 16 — Additional file 16: Supplemental Figure 1. (A) Illustration of the tumor-specific novel SMOC1 isoform detected from the long-read PB_FLIC-Seq workflow showing exon skipping of exons 6 and 7 (highlighted by red box). (B) Sashimi plot generated from tumor and adjacent tissue comparator short-read RNA-Seq data showing exon skipping (highlighted by red box) within tumor SMOC1 expressed transcripts. Supplemental Figure 2. (A) Illustration of the tumor-specific novel CCN2 isoform detected from the long-read PB_FLIC-Seq workflow showing intron retention of introns 2 and 3 (highlighted by light orange box). (B) Sashimi plot generated from tumor and adjacent tissue comparator short-read RNA-Seq data showing splicing events (red box highlights introns of interests) for expressed CCN2 transcripts. [file 12864_2024_10021_MOESM16_ESM.pdf]

Supplemental Information

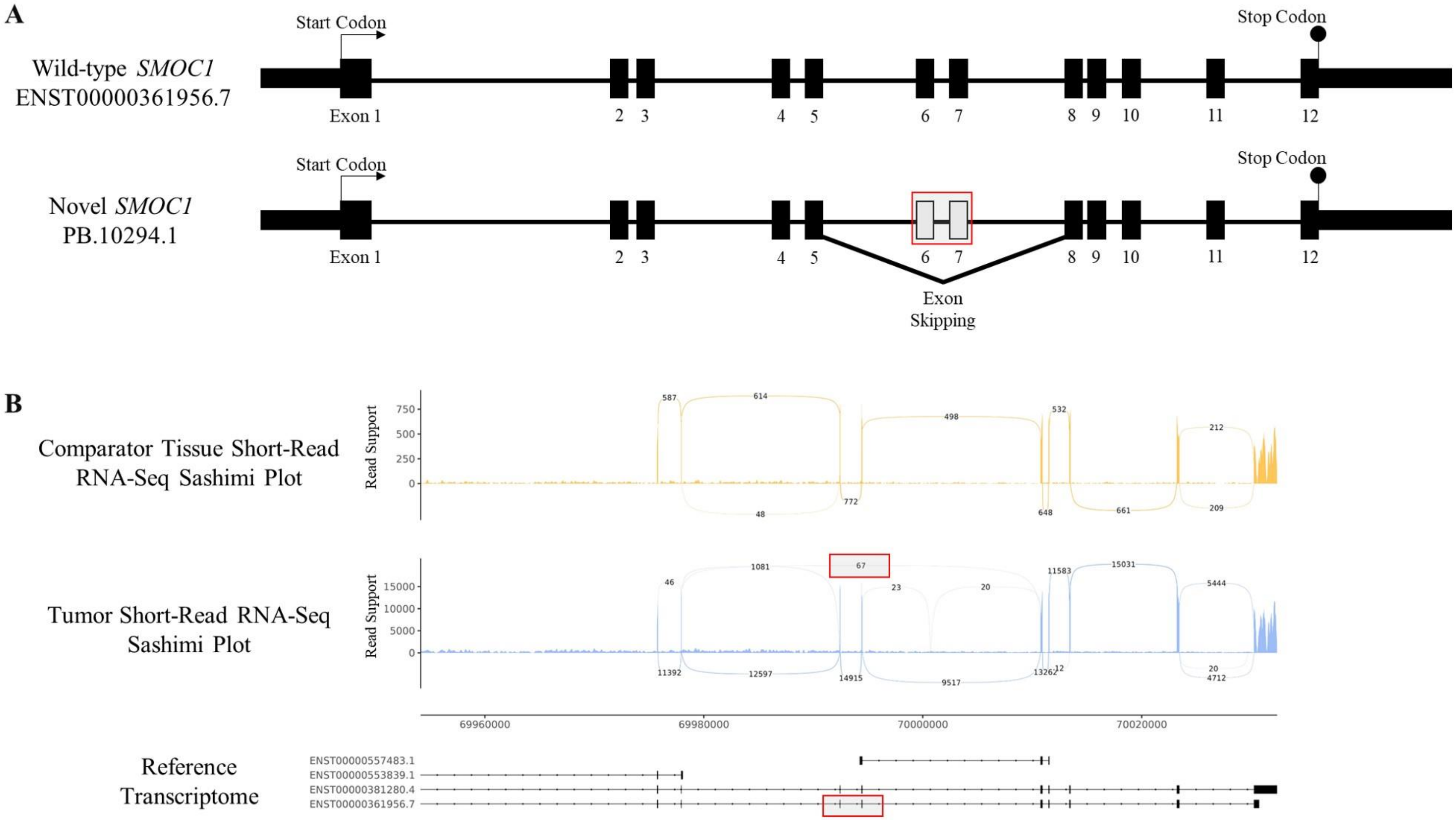

Supplemental Figure 1. (A) Illustration of the tumor-specific novel *SMOC1* isoform detected from the long-read PB_FLIC-Seq workflow showing exon skipping of exons 6 and 7 (highlighted by red box). (B) Sashimi plot generated from tumor and adjacent tissue comparator short-read RNA-Seq data showing exon skipping (highlighted by red box) within tumor *SMOC1* expressed transcripts.

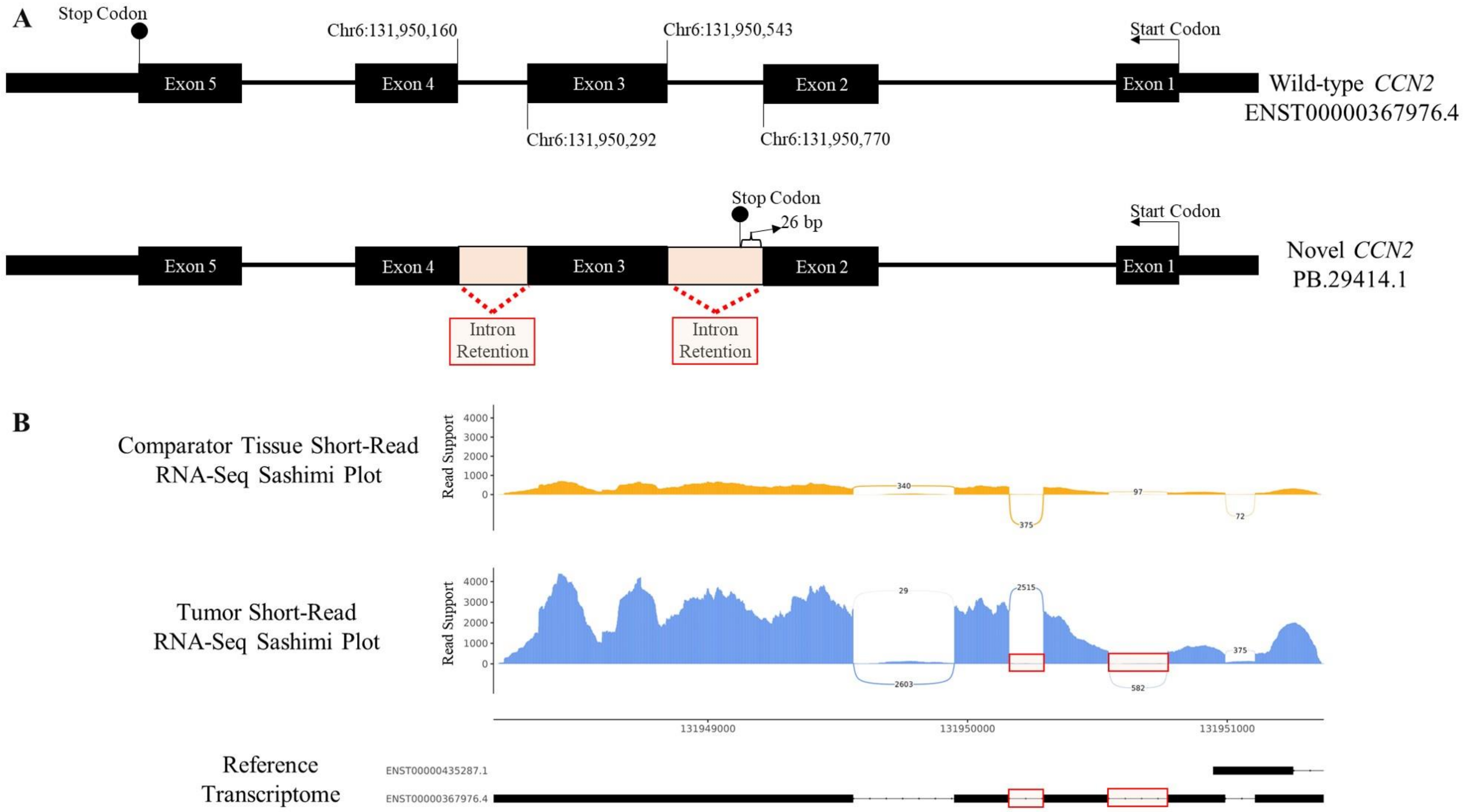

Supplemental Figure 2. (A) Illustration of the tumor-specific novel *CCN2* isoform detected from the long-read PB_FLIC-Seq workflow showing intron retention of introns 2 and 3 (highlighted by light orange box). (B) Sashimi plot generated from tumor and adjacent tissue comparator short-read RNA-Seq data showing splicing events (red box highlights introns of interests) for expressed *CCN2* transcripts.
